# Hypertension and diabetes treatment affordability and government expenditures following changes in patient cost sharing in the “Farmácia popular” program in Brazil: an interrupted time series study

**DOI:** 10.1186/s12889-019-8095-0

**Published:** 2020-01-08

**Authors:** Isabel Cristina Martins Emmerick, Mônica Rodrigues Campos, Rondineli Mendes da Silva, Luisa Arueira Chaves, Andréa Dâmaso Bertoldi, Dennis Ross-Degnan, Vera Lucia Luiza

**Affiliations:** 1 Division of Thoracic Surgery – Department of Surgery, University of Massachusetts Medical School, 67 Belmont street, Worcester, MA 01605 USA; 2 Pharmaceutical Policy Research Fellowship, Department of Population Medicine, Harvard Medical School & Harvard Pilgrim Health Care Institute, Landmark Center, 401 Park Drive Suite 401, Boston, MA 02215 USA; 3 Department of Social Sciences, Sergio Arouca National School of Public Health, Oswaldo Cruz Foundation, 1480 Rua Leopoldo Bulhões #905, Manguinhos, Rio de Janeiro, 21041-210 Brazil; 4 Department of Medicines and Pharmaceutical Services Policies, Sergio Arouca National School of Public Health, Oswaldo Cruz Foundation, 1480 Rua Leopoldo Bulhões #624, Manguinhos, Rio de Janeiro, 21041-210 Brazil; 5 Phamacy Department, Federal University of Rio de Janeiro, Macaé Campus. Av. Aluizio da Silva Gomes, #50, Granja dos Cavaleiros, Macaé, 27930-560 Brazil; 6 Postgraduate Program in Epidemiology, Federal University of Pelotas, Rua Marechal Deodoro 1160, Pelotas, 96020-220 Brazil; 7 Department of Population Medicine, Harvard Medical School & Harvard Pilgrim Health Care Institute, Landmark Center, 401 Park Drive Suite 401, Boston, MA 02215 USA

**Keywords:** Non-communicable diseases, Affordability, Hypertension, Diabetes, Government expenditure, Medicines

## Abstract

**Background:**

Increasing medicines availability and affordability is a key goal of Brazilian health policies. “*Farmácia Popular*” (FP) Program is one of the government’s key strategies to achieve this goal. Under FP, antihypertension (HTN) and antiglycemic (DM) medicines have been provided at subsidized prices in private retail settings since 2006, and free of charge since 2011. We aim to assess the impact of sequential changes in FP benefits on patient affordability and government expenditures for HTN and DM treatment under the FP, and examine their implications for public financing mechanisms and program sustainability.

**Methods:**

Longitudinal, retrospective study using interrupted time series to analyze: HTN and DM treatment coverage; total and per capita expenditure; percentage paid by MoH; and patient cost sharing. Analyzes were conducted in the dispensing database of the FP program (from 2006 to 2012).

**Results:**

FP has increased its coverage over time; by December 2012 FP covered on average 13% of DM and 11.5% of HTN utilization, a growth of over 600 and 1500%, respectively. The overall cost per treatment to the MoH declined from R$36.43 (R$ = *reais*, the Brazilian currency) to 18.74 for HTN and from R$33.07to R$15.05 for DM over the period analyzed, representing a reduction in per capita cost greater than 50%. The amount paid by patients for the medicines covered increased over time until 2011, but then declined to zero. We estimate that to treat all patients in need for HTN and DM in 2012 under FP, the Government would need to expend 97% of the total medicines budget.

**Conclusions:**

FP rapidly increased its coverage in terms of both program reach and proportion of cost subsidized during the period analyzed. Costs of individual HTN and DM treatments in FP were reduced after 2011 for both patients (free) and government (better negotiated prices). However, overall FP expenditures by MoH increased due to markedly increased utilization. The FP is sustainable as a complementary policy but cannot feasibly substitute for the distribution of medicines by the SUS.

## Background

Treatment of hypertension (HTN) and diabetes (DM) is considered a health policy priority in Brazil, with particular attention paid to reducing preventable hospital admissions. Recent reductions have been associated with the expansion of the primary care, which facilitates early detection, and treatment of HTN and DM [1].

The main characteristics of the principal medicines provision mechanisms in place in Brazil are summarized in Table 4 in Appendix. Access to medicines and health care is universal [2] and there is no barrier to obtaining medicines from different sources. Patients can obtain medicines free at public health facilities, through “*Farmácia Popular*” (FP), or by paying out-of-pocket at private retail pharmacies simultaneously.

The “*Farmácia Popular*” (FP) is a medicines provision mechanism first implemented in 2004. In its first phase, medicines were provided in public health care facilities at a flat price, corresponding to the price obtained by the government in open bidding, plus administrative costs. This program was named “*Farmacia Popular Rede Própria*”. In 2006 the program, then named “*Aqui tem Farmacia Popular - AFP”* (“*Farmácia Popular*” is available here- AFP-I), was expanded to private pharmacies contracted with the Ministry of Health. Medicines were charged in a coinsurance model, with the government paying 90% of a reference price and patients paying 10% of the selling price (which might be higher than the reference price). To improve accountability of pharmacies, a new administrative system was implemented in 2009 (AFP-II) that provided information on each patient claim.

Subsequently, antihypertensive, antidiabetic (since 2011), and antiasthma medicines (since 2012) began to be dispensed with zero copayment from patients in both government-owned facilities (*n* = 558) [3] and contracted private pharmacies (*n* = 25,150, covering 63.4% of the 5570 municipalities [3]). This change was named as “*Saúde não tem preço - SNP*” (Health has no price). Except for metformin 500 mg (extended release), all medicines covered in FP for these three conditions that are included in RENAME (the National Essential Medicines List) were provided free-of-charge in SUS. The SNP makes treatment affordable for patients, but this benefit might be unsustainable in the long term, especially in a country that has other forms of provision.

Currently, the FP program continues to provide free medications for treatment of asthma, diabetes and hypertension, and co-finances drugs for treatment of dyslipidemia, osteoporosis, rhinitis, Parkinson’s and glaucoma, as well as contraceptives and geriatric diapers, totaling approximately R$2.9 billion in 2017 [4].

FP is widely seen as a successful program that has expanded coverage in both the number of individuals treated for DM and HTN and in the number of dispensings per person [5–7]. However, a number of studies have cautioned about the program’s high expenditures [8, 9]. On the other hand, the FP program has been associated with reduced rates of hospital admissions and mortality per 100,000 inhabitants [4]. In parallel with increased government expenditures, families’ health expenditures have also been increasing [10], especially for medicines. It is thus relevant to examine the impact of FP on both government expenditures and medicines affordability.

This paper aims to analyze the impact of the sequential *Farmácia Popular* interventions on patient affordability and government expenditures for HTN and DM treatment under the FP program, and consider their implications for public financing mechanisms and program sustainability.

## Methods

This is a longitudinal, retrospective study using interrupted time series (ITS) to examine out-of-pocket payments and MoH expenditures for HTN and DM treatment. The main outcomes addressed are rates of HTN and DM treatment coverage, number of individuals in FP, total expenditures, percentages paid by MoH, treatment cost per capita and out of pocket payment.

The Brazilian National Ethics Committee, by the National School of Public Health – Fiocruz – Brazil and the WHO ERC, approved the ISAUM-Br project, which is the basis for this paper.

### Interventions

The study interventions are two changes in patient cost sharing in AFP. The April 2009 AFP-II policy involved a reduction in reference prices for most FP medicines by an average of 24.5%, coupled with administrative changes aiming to improve accountability. In February 2011, the “*Saúde não tem preço*” (SNP) program was implemented, under which all covered medicines for HTN and DM were dispensed free of charge to patients. FP private pharmacies were reimbursed according to a set of negotiated prices, while in government-owned pharmacies, medicines were fully subsidized. Only FP private pharmacies are addressed in this paper.

### Data source and study population

The FP information system is the first widespread governmental administrative system on medicines dispensing in Brazil. The FP information system in contracted pharmacies is managed by the Unified Health System Informatics Department (DATASUS). Data include patient unique identification number (CPF), price paid, date of purchase, prescribed daily dose and amount procured. CPF allows linking to data on gender and date of birth. In the majority of cases, the buyer corresponds to the patient for patients over 18 years old. Other administrative systems cover a small set of medicines (e.g. ARVs, high cost medicines) and are not integrated at national level.

FP program eligibility criteria have remained unchanged during the program: all medicines are sold only if a national ID and a valid prescription are presented. During the study period medicines were dispensed on a monthly basis, although prescriptions were valid for 120 days. Over time, the number of participating private sector pharmacies expanded substantially, especially in some regions [3].

Data are derived from an electronic point-of-sales dispensing program implemented in 2006 in FP retail pharmacies and then integrated online by DATASUS. Available data include patient and pharmacy identifiers, patient age and gender, geographic location of the pharmacy, date of dispensing, name and quantity of medicine dispensed, daily prescribed dose, amount of MoH reimbursement, and patient copayment.

We use data on dispensing of HTN and DM medicines from October 2006 to December 2012. All patients with at least one dispensing during the study period were included in this analysis. Dispensing data are of good quality and relatively complete, with duplicate cases accounting for less than 0.005% and individual-level missing data at less than 0.05%. We excluded encounters with missing data on any outcome variables from all analyses.

Medicines covered by the program include four oral antidiabetic medications (glibenclamide 5 mg, and metformin 500 mg, 850 mg, and slow release 500 mg formulations), insulin NPH and regular and six antihypertensive medications (atenolol 25 mg, propranolol 40 mg, hydrochlorothiazide 25 mg, captopril 25 mg, enalapril 5 mg, and losartan 50 mg).

### Analysis

We analyzed five study outcomes related to FP *program coverage*, *MoH expenditures*, and *affordability*, as follows:

1) Monthly number of individuals who received at least one dispensing at AFP pharmacies;

2) Total monthly program expenditure in *reais* (Brazilian currency), including total MoH expenditure and total patient payments;

3) Monthly percentage of expenditure paid by the MoH;

4) Monthly expenditure per treatment (per capita), which is the total monthly expenditure divided by the number of individuals in the program; and.

5) Average monthly out-of-pocket payment, which is the average amount paid by patients per treatment.

Annual inflation was a relatively stable 3 to 7% during the study period. We performed a monthly inflation correction for all financial outcomes [11]. We report all financial outcomes in 2012 inflation-adjusted Brazilian *reais*; the exchange ratio during the study period was roughly 2 Brazilian *reais* to 1 US dollar [12].

As an indicator of potential *program sustainability*, we estimated the level of expenditure that would be needed to fully cover all individuals in Brazil with DM and HTN through the FP program, and calculated the percentage that would represent of total MoH expenditures on medicines, yearly from 2006 to 2012.

It has been demonstrated that most people with HTN and DM diagnoses, respectively 95 and 85%, are under pharmacological treatment in Brazil [13]. Thus, it seems fair to use national prevalence to estimate potential FP costs, assuming that all patients were treated through the program. The costs per individual treated in the program consider the average cost per capita per type of disease HTN or DM.

To create this sustainability measure, we first developed two measures estimating annual FP program utilization: a) Number of unique individuals with at least one dispensing within a given year; b) Average number of individuals receiving at least one dispensing per month, averaged across 12 months in a given year (i.e., allowing individuals to repeat across months). We used these to construct annual and monthly estimates of program coverage, where the denominator of each measure is an estimate of the annual prevalence of each disease in Brazil, as a *proxy* for the number of individuals who should be under treatment [14].

In addition to deriving yearly coverage estimates using FP program data, we also used the FP coverage estimates reported in the following surveys: National Program for Improving Access and Quality of Primary Health Care *(Programa Nacional de Melhoria do Acesso e da Qualidade da Atenção Básica* - PMAQ-AB) [15], Brazilian Survey on Medicine Access, Utilization and Rational Use of Medicines (*Pesquisa Nacional sobre acesso e utilização e promoção do Uso Racional de Medicamentos* – PNAUM) [13], Surveillance of risk-factor for chronic diseases through telephone interviews (*Vigilância de fatores de risco e proteção para doenças crônicas por inquérito telefônico* – VIGITEL [14] and National Health Survey (Pesquisa Nacional de Saúde – PNS) [16].

We did not adjust the monetary values used in this analysis for inflation, since we are comparing the proportions of expenditures in each year, and not actual expenditures themselves.

### Statistical methods

To analyze the impact of *Farmácia* Popular interventions on affordability and MoH expenditures, we used ITS segmented linear regression models to determine the effect of the FP policy changes on the study outcomes. In estimating effects, ITS models adjust for pre-existing trends in the period before the policy change [17]. Segmented linear regression models were constructed using the “*prais , corc”* command in STATA v12 [18], we analyzed linearity and autocorrelation. ITS considered to be one of the strongest quasi-experimental design to evaluate longitudinal effects of interventions, while segmented regression analysis is a commonly used statistical method for estimating intervention effects in ITS studies [17–22].

Our ITS models included three segments, baseline and one for each of the two program periods, with 29, 22, and 23 monthly observations, respectively. The segmented regression model was specified as follow [17, 20]:

$$ {Y}_t={\beta}_0+{\beta}_1\ast {month}_t+{\beta}_2\ast {AFP II}_t+{\beta}_3\ast months\ {after\ AFPII}_t+{\beta}_4\ast {AFP}_t+{\beta}_5\ast months\ after\ {SNP}_t+{e}_t $$

In this model, time (t) is a continuous variable indicating time in months from the start of the observation period; Y_t_ = outcome variable in month t; β_0_ = level at the start of the observation period (intercept); β_1_ = baseline trend; month_t_ = number of months from start of observation; AFPII_t_ = whether month t is after AFPII; β_2_ = level change after the AFPII; β_3_ = trend change after the AFPII; SNP_t_ = whether month t is after SNP; β_4_ = Level change after the SNP; β_5_ = trend change after the SNP; e_t_ = residual error.

The baseline segment was fit with an intercept and a variable estimating trend. We estimate each policy effect by a variable representing the change in level of the outcome immediately after the policy and a second representing the change in trend of the post-policy segment. Patients would experience changes in copayment only when they presented to fill a prescription after the policy change. We thus defined a post-policy implementation period of 2 months for the program to take effect; these periods were excluded in the ITS models so that we could estimate stable post-intervention effects. Additionally, we performed a sensitivity analysis considering the possibility of autocorrelation, assessing the significance of the Durbin-Watson statistic. We found that all outcomes have some level of autocorrelation, we compare the use of “prais” alone, “prais, var rhotype (dw)”, and “prais var, corc” [18]. We made an option to use Cochrane-Orcutt procedure “prais var., corc” since it presented the better adjustment. The sensitivity analysis showed that small autocorrelation did not impact the direction, significance of the findings. (Additional file 1).

We retained all parameters in the models regardless of statistical significance. We highlight the results with *p* < 0.05. To create single number summaries of policy effects, we calculated estimates of the relative changes in outcomes compared to expected values based on prior trends in April 2010 and February 2012, about 1 year after the two copayment interventions.

## Results

A total of 6,059,643 and 14,447,006 patients received medicines for DM or HTN, respectively, from the FP program. The mean age was 55 years for diabetes and 56 for hypertension patients, with females comprising about 60% of patients for both diseases. The southeast region represented the majority of patients in the program (Table 1) (Additional file 2).

**Table 1:** Participants in “Farmácia Popular is Available Here” program by gender, age, region, and specific coverage. Brazil, 2006 to 2012

Variables	Diabetes		Hypertension	
Age (n; mean (SD))				
Total	6,059,643	55 (15)	14,447,006	56 (15)
Gender (n, %)^a^				
Female	3,618,239	59.7%	8,666,405	60.0%
Male	2,425,635	40.0%	5,738,940	39.7%
Region (n, %)				
North	169,525	2.8%	470,286	3.3%
Northeast	852,271	14.1%	2,127,680	14.7%
Southeast	3,790,268	62.5%	8,333,436	57.7%
South	887,754	14.7%	2,566,954	17.8%
West-Center	359,825	5.9%	948,650	6.6%
Coverage^1^	Number of individuals in the FP	Annual coverage	Number of individuals in the FP	Annual coverage
2006	186,286	3.2%	348,903	1.6%
2007	746,279	11.2%	1,554,871	6.1%
2008	1,251,049	16.7%	2,711,688	9.7%
2009	1,300,919	17.4%	2,765,155	9.8%
2010	1,041,056	12.9%	2,039,368	7.1%
2011	2,682,000	33.2%	7,008,960	24.3%
2012	3,755,010	40.6%	9,487,841	32.6%
Coverage^2^	Average individuals per year	Average Monthly coverage	Average individuals per year	Average Monthly coverage
2006	52,004	0.9%	101,945	0.5%
2007	150,409	2.3%	320,338	1.2%
2008	280,316	3.7%	670,006	2.4%
2009	290,382	3.9%	667,345	2.4%
2010	254,860	3.2%	538,718	1.9%
2011	715,403	8.9%	1,722,162	6.0%
2012	1,200,509	13.0%	3,330,403	11.5%

Coverage 1. Numerator is the number of individuals with at least one dispensing within the year (individuals do not repeat)

Coverage 2. Numerator is the average monthly individuals in each year (sum of individuals in each month within 1 year divided by 12 months) (the same individual can be count in different months in the same year)

For both coverage indicators the denominator is the estimate prevalence for each disease, which means the number of individuals that should be under treatment. (Brazilian Basic Indicators - http://tabnet.datasus.gov.br/cgi/idb2012/matriz.htm)

^a^Gender missing Diabetes 0.26% Hypertension 0.29%

### FP coverage

Annual coverage of unique patients varied from 3.2 to 16.7% for DM and 1.6 to 9.7% for HTN, from 2006 to 2008, while average monthly coverage varied from 0.9 to 3.7% for DM and 0.5 to 2.4% for HTN, respectively. In 2009 and 2010, just after the AFP-II, coverage of unique patients decreased from 17.4 to 12.9% for DM and 9.8 to 7.1% for HTN, while average monthly coverage decreased from 3.9 to 3.2% for DM and 2.4 to 1.9% for HTN, respectively. After SNP, there was an impressive increase in coverage of unique patients that reached 40.6% for DM and 32.6% for HTN by the end of follow-up, while average monthly coverage reached 13.0 and 11.5%, respectively (Table 1).

### Number of individuals in FP

**Fig. 1:**
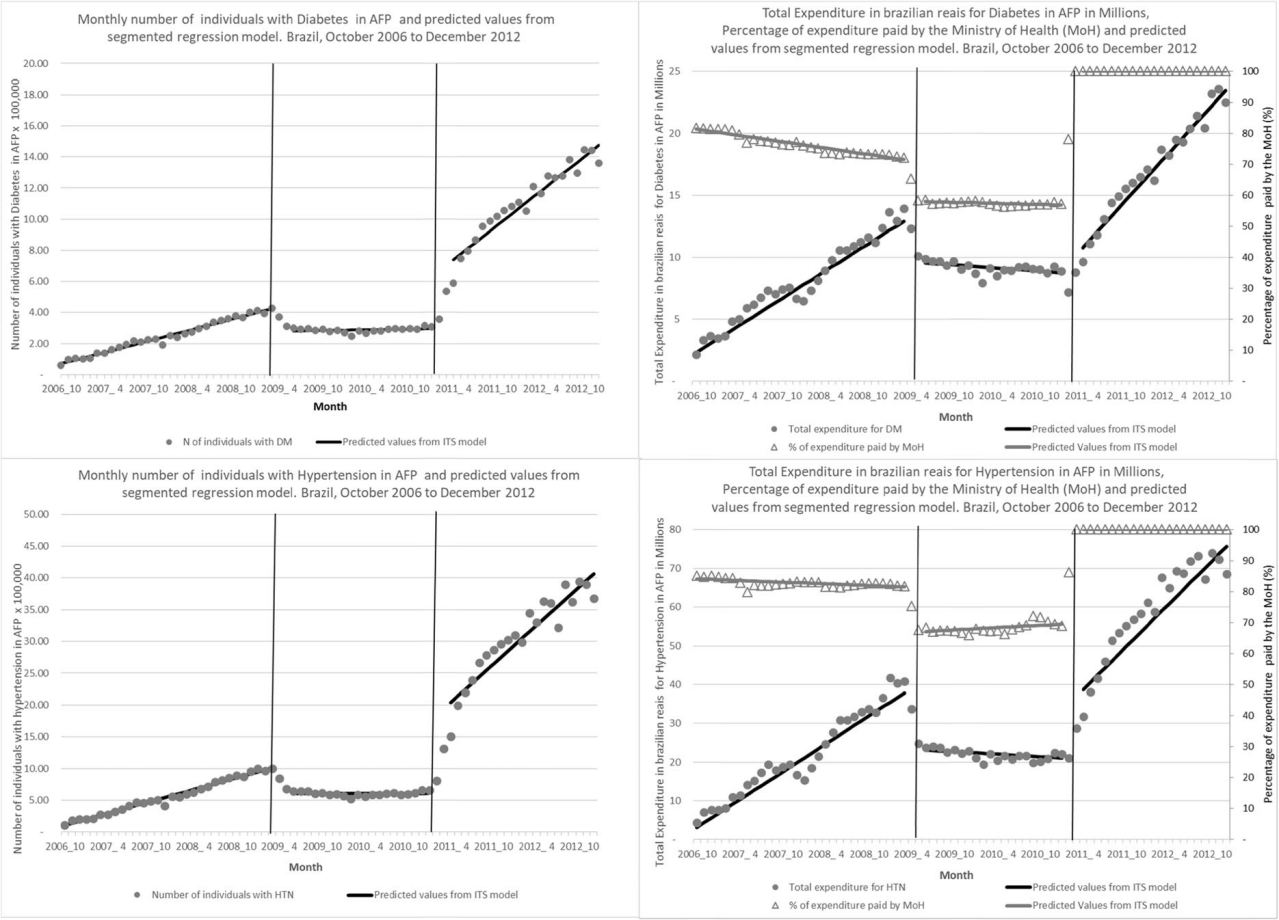
Number of individuals and total expenditures in FP and percentage paid by the MoH, and predicted values from segmented regression models for diabetes and hypertension, by stage of the Farmácia Popular program, Brazil, 2006 to 2012

During the baseline period prior to the cost sharing changes, the numbers of individuals covered by the FP program were about 60 and 73 thousand for DM and HTN, respectively, with increasing trends of 12 and 31 thousand additional individuals per month. The AFP-II policy changes were associated with a significant decrease in level and trend, resulting in a relative decrease of over 70% for DM and 85% for HTN diseases by April 2010 (Table 2 and Fig. 1). The free medicines policy under SNP was associated with a large expansion in FP participation. The relative increases by February 2012 were 615 and 1507% for DM and HTN, respectively (Table 2 and Fig. 1).

**Table 2:** Baseline level and trend in monthly number of individuals per 100,000, MoH total expenditure^c^, expenditure per treatment percapita^c^, and out of pocket payment^c^ for diabetes and hypertension, and changes in level and trend by stage of the Farmácia Popular program, Brazil, 2006 to 2012

	Baseline			AFP II (April 2009)			SNP (February 2011)			
	Level	Trend	Mar-09	Level AFP II Change at the intervention (95% CI)	Trend AFP II (95% CI)	% relative change - AFP II April 2010	Jan-11	Level SNP Change at the intervention (95% CI)	Trend SNP (95% CI)	% relative change SNP February 2012
Diabetes										
Number of individuals^a^	0.60	0.12	2.42	**−1.74** **(− 2.19 to − 1.29)**	**− 0.11** **(− 0.14 to − 0.08)**	**−73.6**	1.16	**4.58** **(4.09 to 5.08)**	**0.32** **(0.28 to 0.36)**	**615.6**
Total expenditure^b^	2.12	0.36	7.47	**−4.42** **(− 7.55 to − 1.29)**	**− 0.37** **(− 0.58 to − 0.15)**	**−69.9**	3.55	2.9 (− 0.35 to 6.16)	**0.5** **(0.3 to 0.8)**	**260.6**
Percentage MoH	81.33	− 0.32	76.47	**−13.93** **(− 15.87 to − 11.99)**	**0.33** **(0.2 to 0.46)**	**−14.3**	61.98	**42.87** **(40.85 to 44.89)**	0 (− 0.16 to 0.16)	**69.0**
Expenditure per treatment (percapita in reais)	33.07	−0.11	31.41	5.97 (−6.82 to 18.75)	−0.2 (−1.03 to 0.62)	12.4	30.88	**−15.83** **(− 28.67 to − 2.99)**	0.49 (− 0.5 to 1.49)	**−38.8**
Out of pocket payment	6.25	0.07	7.35	**6.64** **(4.0 to 9.27)**	**−0.21** **(− 0.38 to − 0.03)**	**52.4**	11.44	**−12.27** **(− 14.95 to − 9.59)**	0.13 (− 0.08 to 0.34)	**− 111.3**
Hypertension										
Number of individuals^a^	0.73	0.31	5.42	**−4.88** **(−6.63 to − 3.12)**	**−0.3** **(− 0.42 to − 0.17)**	**− 86.0**	1.46	**15.5** **(13.59 to 17.41)**	**0.85** **(0.7 to 1.01)**	**1507.4**
Total expenditure ^b^	0.20	1.27	19.27	**−19.4** **(− 36.3 to − 2.4)**	**−1.3** **(−2.4 to − 0.2)**	**− 93.3**	2.38	**34.6** **(17.5 to 51.8)**	0.74 (− 0.6 to 2.08)	**1812.6**
Percentage MoH	83.41	−0.05	82.65	**−16.4** **(− 18.8 to − 14)**	**0.3** **(0.1 to 0.5)**	**−16.2**	70.83	**29.5** **(26.9 to 32)**	− 0.2 **(− 0.4 to 0)**	**36.4**
Expenditure per treatment (percapita in reais)	36.43	0.01	36.59	4.51 (−17.33 to 26.35)	−0.4 (− 1.81 to 1.01)	0.3	33.34	**−14.6** **(− 36.49 to 7.3)**	0.44 (− 1.26 to 2.13)	**−34.6**
Out of pocket payment	6.29	0.01	6.41	**7.63** **(4.04 to 11.21)**	−0.21 (− 0.45 to 0.02)	**80.8**	9.94	**−9.66** **(−13.32 to − 5.99)**	0.21 (− 0.08 to 0.49)	**− 101.7**

Significant values *p* < 0.05 are highlighted in bold

^a^The number of individuals was divided by 100,000

^b^The total expenditures was divided by 1.000.000

^c^All monetary values are expressed in Brazilian reais and the monetary values were adjusted by inflation to December 2012

*CI* Confidence interval

### Total expenditure for DM and HTN

Total expenditures in the program tracked the changes observed in the number of individuals participating for both DM and HTN, with expenditures increasing steadily prior to April 2009, then experiencing relative declines of 69.9 and 93.3% by April 2010, 1 year after AFP-II implementation, for DM and HTN, respectively (Table 2 and Fig. 1). The free medicines SNP policy was associated with relative increases in total FP expenditure of 260 and 1812% for DM and HTN, respectively (Table 2 and Fig. 1).

### Percentage of expenditures by the Ministry of Health

Prior to 2009, the MoH was responsible for 81.3 and 83.4% of the total expenditures for DM and HTN medications in the program, with a slightly decreasing trend. The cost sharing changes introduced by the AFP-II policy reduced the MoH share of expenditures at 1 year after the policy change by 14.3 and 16.2% for DM and HTN, respectively (Table 2 and Fig. 1). Following the SNP free medicines policy, the MoH started to cover 100% of medicines expenditures, representing an increase of 69% for DM and 36% for HTN by February 2012 (Table 2 and Fig. 1).

### Per capita expenditure for DM and HTN treatment

**Fig. 2:**
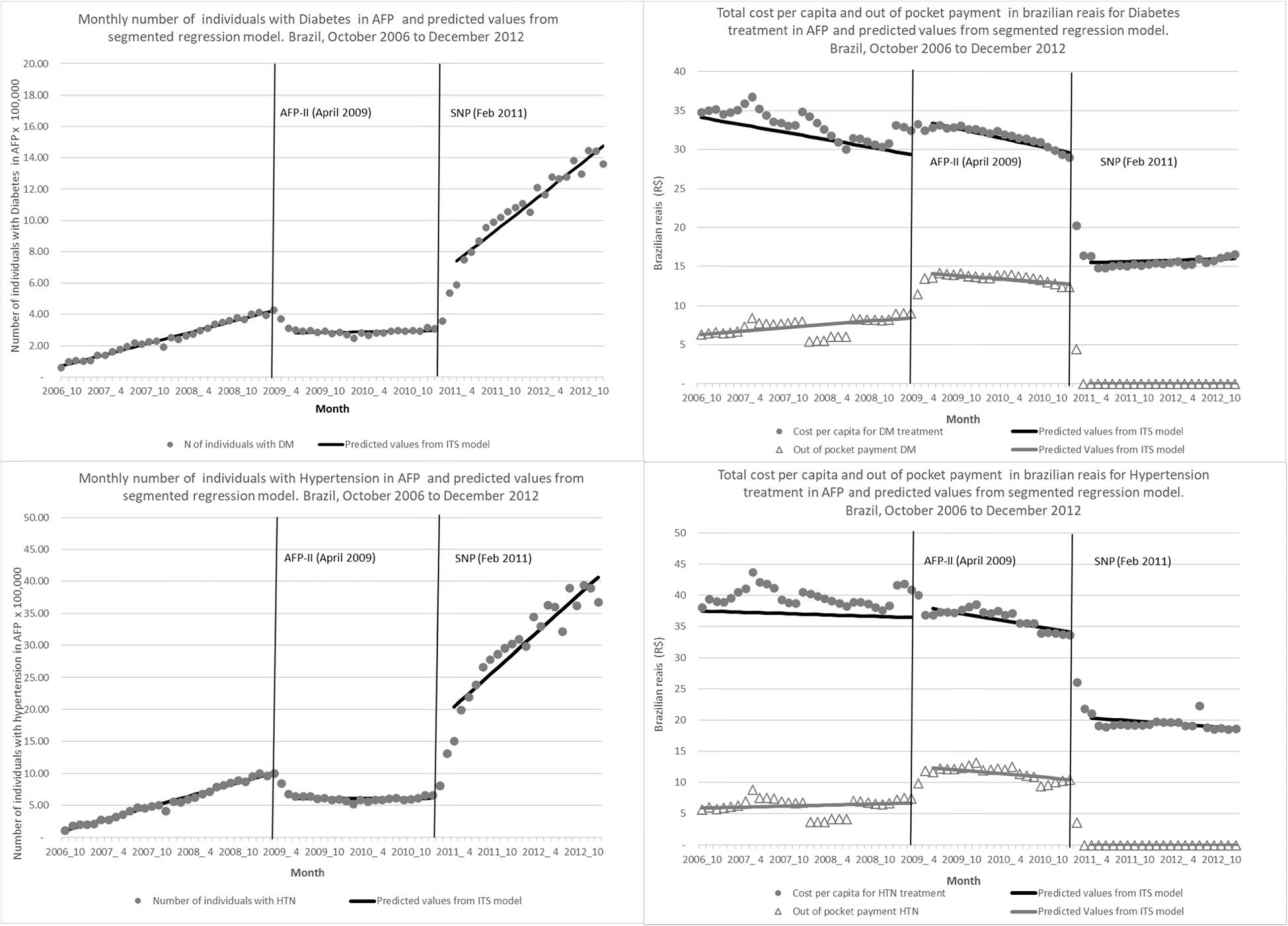
Number of individuals and treatment cost per capita and out of pocket payment, and predicted values from segmented regression models for diabetes and hypertension, by stage of the Farmácia Popular program, Brazil, 2006 to 2012

The cost per treatment per person prior to 2009 varied from R$33.1 to R$31.4 to and R$36.4 to 36.6 for DM and HTN, respectively, with no significant change following the AFP-II policy change. The free medicines SNP policy was associated with a decrease of around R$15 *per treatment*, representing a reduction over 37% by February 2012 for both diseases (Table 2 and Fig. 2).

### Out of pocket payment

In 2006, patients paid an average of R$6.3 for their monthly DM and HTN treatment, respectively. The AFP-II policy change was associated with an increase in out of pocket payment of R$6.6 for DM and R$7.6 for HTN at the time of the intervention, with a relative increase after 1 year of 52.4 and 80% for DM and HTN, respectively (Table 2 and Fig. 2). The full subsidy introduced by SNP made medicines available free of charge to patients after 2011.

### Estimate of *Farmácia Popular* program sustainability

Based on the varying prevalence estimates in national surveys, the percentage of total MoH expenditures on medicines that would be required to treat all patients under the free medicines policy would vary from 4.9 to 18.7% for DM, and from 23.1 to 72.1% for HTN patients, respectively (Table 3).

**Table 3:** Annual coverage and sustainability estimated for Farmácia Popular Program, (FP, PMAQ, PNAUM,VIGITEL,PNS), Brazil 2007 to 2015

Year	Prevalence^a^	Coverage (%)						Total MoH expenditures on medicines (in Millions of reais)^h^	Estimated Expenditure to cover all people by Farmácia Popular (as a percentage of total MoH expenditures on medicines)					
		FP_Cov_1^b^	FP_Cov_2^c^	PMAQ^d^	PNAUM^e^	VIGITEL^f^	PNS^g^		FP_Cov_1	FP_Cov_2	PMAQ	PNAUM	VIGITEL	PNS
Diabetes														
2007	9.3	11.2	2.3	–	–	–	–	5176.04	9.3	46.1	–	–	–	–
2008	10.3	16.7	3.7	–	–	–	–	5866.20	8.5	37.8	–	–	–	–
2009	10.0	17.4	3.9	–	–	–	–	6765.46	8.5	38.0	–	–	–	–
2010	10.4	12.9	3.2	–	–	–	–	6988.75	10.3	42.0	–	–	–	–
2011	10.3	33.2	8.9	–	–	16.7	–	8348.67	4.9	18.5	–	–	9.8	–
2012	11.7	40.6	13.0	16.2	–	23.1	–	9656.00	6.0	18.7	15.0	–	10.5	–
2013^i^	10.9	52.9	21.2	–	18.3	24.8	57.4	11,467.11	5.5	13.7	–	15.8	11.7	5.1
Hypertension														
2007	35.8	6.1	1.2	–	–	–	–	5176.04	42.2	205.0	–	–	–	–
2008	38.4	9.7	2.4	–	–	–	–	5866.20	39.2	158.7	–	–	–	–
2009	37.7	9.8	2.4	–	–	–	–	6765.46	40.7	168.8	–	–	–	–
2010	36.8	7.1	1.9	–	–	–	–	6988.75	43.9	166.2	–	–	–	–
2011	36.8	24.3	6.0	–	–	16.1	–	8348.67	23.1	94.0	–	–	37.9	–
2012	36.8	32.6	11.5	15.3	–	22.8	–	9656.00	25.3	72.1	54.2	–	36.2	–
2013^i^	36.5	39.2	15.1	–	16.0	20.9	35.9	11,467.11	25.1	65.2	–	61.5	47.0	27.4

a. prevalence in the population 35 years old and older. VIGITEL (Brazilian Basic Indicators - http://tabnet.datasus.gov.br/cgi/idb2012/matriz.htm)

^b^Coverage 1. Estimated coverage in FP - Numerator is the number of individuals with at least one dispensing within the year (individuals do not repeat)

^c^Coverage 2. Estimated coverage in FP Numerator is the average monthly individuals in each year (sum of individuals in each month within 1 year divided by 12 months) (the same individual can be count in different months in the same year)

^d^PMAQ = National Program for Improving Access and Quality of Primary Health Care (PMAQ); source: http://dab.saude.gov.br/portaldab/ape_pmaq.php

^e^PNAUM = Brazilian Survey on Medicine Access, Utilization and Rational Use of Medicines. Source: (Brasil. Ministério da Saúde. Secretaria de Ciência, Tecnologia e Insumos Estratégicos em Saúde. Departamento de Assistência Farmacêutica e Insumos Estratégicos et al., In press)

^f^VIGITEL = Surveillance of risk-factor for chronic diseases through telephone interviews; Source (Brasil. Ministério da Saúde. Secretaria de Vigilância em Saúde. Secretaria de Ciência, Tecnologia e Insumos Estratégicos, 2015)

^g^PNS = National Health Survey; Source: (Sarmento Costa et al., 2016)

^h^Ministry of Health – national accounts (Rondineli et al., 2016)

^i^2013 AFP coverage is estimated based on coverage linear regression after February 2011

## Discussion

In this paper, we used secondary data from the FP program and data from several national surveys to estimate utilization, government expenditures, and patient out of pocket payments, for DM and HTN treatment and then consider these results in the context of FP sustainability. This unique combination of information is not common in low- and middle-income countries.

The FP program co-exists with the free-of-charge medicines supply system in public health facilities, both of which are governmental mechanisms to provide medicines to patients with chronic illness. We studied antihypertension and hypoglycemic treatment, available free-of-charge in both programs, in order to understand FP program costs and sustainability.

For both diseases, the interventions were associated with similar patterns of change for the outcomes analyzed. The reduction in reference prices in 2009, together with administrative changes (AFP-II), resulted in an increase in patient cost sharing and associated drop out of users. The SNP, on the other hand, resulted in the opposite effects on these measures.

Affordability, as measured by level of patient out-of-pocket payment, decreased after AFP-II but improved substantially when medicines were dispensed for free following the SNP policy. Considering that, prior to the FP program, patients paid the full price of medicines out-of-pocket at retail pharmacies, the simple existence of a government subsidy mechanism increased overall affordability over time. This is important since it has been shown that there is a high burden of medicines expenditures at household level in Brazil [10], despite free-of-charge medicines dispensing in SUS. Since private pharmacies are much more widespread than government health facilities and open for longer hours, this represented an improvement for patients in both affordability and convenience.

The effects of the reduction of reference prices following the AFP-II policy change have already been discussed in other publications [23, 24]. More restrictive enforcement measures were implemented, aimed at reducing corruption [25] and improving control and audit mechanisms [23], but this may have impaired program use by patients, judging by reductions in the number of individuals using the program. Not surprisingly, the SNP free medicines policy had the opposite effect, increasingly attracting users to FP.

Overall, the policy changes under AFP-II showed deleterious effects from both the government and patient perspectives. Despite reducing MoH overall expenditures by reducing their level of cost sharing, the savings were proportional to the number of individuals still using the program. There were no changes in efficiency, since there were no significant reductions in expenditure per treatment. After implementation of the free medicines SNP policy, overall MoH expenditures increased significantly because of increased coverage. However, the cost per treatment was about 40% more efficient, so the relative change in expenditures after 1 year of SNP was 700%, despite a 1600% increase in the number of individuals treated. This improvement in efficiency may be related to gains from economies of scale in drug purchasing and substitution of generic drugs [26]. We previously found a growth of 20% in generic antihypertensive medicines use after SNP [27] as well as evidence of a 75% growth in sales volume for DM and HTN medicines covered in FP 2011 and 2012 [6].

We found large discrepancies in estimates of the size of the DM and HTN populations and FP coverage in the literature [14, 15, 28–30], so we estimated coverage using two different methods. Our estimates for coverage of number of unique patients align with PMAQ, PNAUM, VIGITEL, while PNS is similar to our estimates of average number of monthly patients covered. If we assume that all DM and HTN patients would choose to obtain their medicines through FP private pharmacies, the program would consume in 2013 from 30.6 to 79% of the total MoH medicines budget for treating those two diseases alone. In the same year, the total estimated spending for primary health care medicines provided free-of-charge in public health facilities was R$2.26 billion [31]. As argued previously [6], since private pharmacies are reimbursed according to prescriptions filled and either public or private sector prescriptions are accepted, this poses important concerns regarding FP sustainability.

Considering the estimated financial outlay that would be required for MoH to cover all HTN and DM patients in the country through FP, it is likely that the program is sustainable only as a complementary policy and not as a substitute to distribution of medicines by the SUS. This is in accordance with the program’s original purpose, which was to cover those low-income individuals with key chronic illnesses who used private health care services but could not afford medicines. However, due largely to deficiencies in the SUS, the FP came to be used as a substitute channel for provision of medicines [6]. Despite its positive impact on access, utilization, and affordability, it is important to consider financial sustainability when assessing the value of different medicines provision mechanisms in the country.

The study limitations include having no data on medicines that are not part of the FP, making it impossible to evaluate the impact of FP policies on the utilization of other medicines used to treat diabetes and hypertension. Furthermore, since we use data from surveys to estimate program coverage, we need to also consider the limitations of these sources of secondary data.

Individuals are allowed to be in multiple treatments. Per-capita cost was considered the total cost of all medicines covered in the month divided by the number of individuals in treatment under FP. As we used data at individual level, we think this is a reasonable approach, assuming that the distribution of medicines users would be similar in SUS and FP.

Despite increased investment over time in SUS strengthening, there was not any specific new policy or intervention change that occurred at the same point in time of the studied interventions [6, 12]. Therefore, since we controlled for pre-existing trend and the small autocorrelation addressed in the analysis, we can infer that the observed changes estimated in this paper are related to the interventions and not to another confounding policy.

## Conclusion

The FP greatly increased its coverage during the period analyzed and substantially improved the affordability of chronic illness treatment when medicines for diabetes and hypertension became free-of-charge in 2011. There was also an improvement in government expenditure efficiency, since the treatment cost per capita declined after 2011 due to purchasing leverage. However, overall MoH expenditures in the FP program increased substantially because of the increased number of users. FP appears to be sustainable only as a complementary policy, not as replacement for the SUS provision of medicines at primary healthcare facilities. Considering the magnitude of the FP program in the Brazilian pharmaceutical market, future studies should address the optimum treatment cost per capita*,* especially in the context of the Brazilian economic and political crises.

### Supplementary information

**Additional file 1.** Analysis Syntax - Diabetes and Hypertension interrupted time series models.

**Additional file 2.** Diabetes and Hypertension interrupted time series models - original, analyzed data and graphs.

## Data Availability

The raw data is not available in the manuscript since the main database used is protected and not open access. However, the datasets used and/or analyzed during the current study [and its supplementary information files] are available from the corresponding author on reasonable request.

## Appendix

**Table 4:** Main characteristics of the principal medicines provision system in place in Brazil. 2015

Aspect	Medicines Provision System			
	Farmácia Popular – government owned dispensing facilities	Farmacia Popular – covenantal private pharmacies	Public health care dispensing facilities	Out-of-pocket (private pharmacies and outlets)
Management	Oswaldo Cruz Foundation (Fiocruz)	Department of Pharmaceuticals – Ministry of Health	Decentralized, mostly at Municipality level	Private
Dispensing / selling facilities	Managed by government organizations (e.g. Municipality Health Secretariats, University Hospitals, NGOs), covenant with Fiocruz	Private pharmacies covenanted with the MoH	Public dispensing facilities, generally located inside health care facilities	Private pharmacies and pharmaceutical outlets
Procurement	Managed by Fiocruz through open bid	Private pharmacies	Each government level. Primary health care medicines are mostly procured at Municipality level, except medicines to endemic diseases, which are central procured by the MoH	Private pharmacies
Reference list	Yes, 41 therapeutic groups, 117 items	Yes, 9 therapeutic groups, 117 items	Yes, NEML (Rename)	No
Clients/ prescription	Anyone	Anyone	Generally, only prescriptions from a public health facility is accepted	Anyone
Payment	Fixed price antihypertensive, antidiabetics and antiashmatic medicines are free-of-charge to patients since 2011	90% of reference price paid by the government, difference paid by the patient (it occurs that selling price is bigger than reference price). 100% is paid by the government for antihypertensive, antidiabetics and antiasthmatic medicines since 2011	Free-of-charge to patients	Out-of-pocket Maximum retail price defined by the regulatory agency (Anvisa)

Source: Adapted from Silva, 2014 [32]

## Abbreviations

AFP: *Aqui tem Farmácia Popular*- “*Farmácia Popular*” is available here
DM: Diabetes Melitus
FP: *Farmácia Popular*
HTN: Hypertension
MoH: Ministry of Health
SNP: Saúde não tem preço – SNP -Health has no price
WHO ERC: World Health Organization
