# Supplementary material for: Hypertension and diabetes treatment affordability and government expenditures following changes in patient cost sharing in the “Farmácia popular” program in Brazil: an interrupted time series study
Source: BMC Public Health. 2020 Jan 8;20:24. doi: 10.1186/s12889-019-8095-0 (PMC6951004; doi:10.1186/s12889-019-8095-0)
Supplement: Supplementary file 1 — Additional file 1. Analysis Syntax - Diabetes and Hypertension interrupted time series models. [file 12889_2019_8095_MOESM1_ESM.pdf]

```
  ___  ____  ____  ____  ____ (R)
 /__    /   ____/   /   ____/
___/   /   /___/   /   /___/   12.1   Copyright 1985-2011 StataCorp LP
  Statistics/Data Analysis            StataCorp
                                      4905 Lakeway Drive
     Special Edition                  College Station, Texas 77845 USA
                                      800-STATA-PC        http://www.stata.com
                                      979-696-4600        stata@stata.com
                                      979-696-4601 (fax)

Single-user Stata network perpetual license:
       Serial number:  93611859953
         Licensed to:  Stata
                       BioStat

Notes:
      1.  (/v# option or -set maxvar-) 5000 maximum variables

  1 . doedit D:\Dropbox\ISAUM-Br_Paper_OOP\BMC-Public_Health\BMC-Public_Health_rev-2\Paper_O
    > OP_sintax_Ago_02_2019.do

  2 . use "D:\Dropbox\ISAUM-Br_Paper_OOP\BMC-Public_Health\BMC-Public_Health_rev-2\HTN_DM_OO
    > P_Jul_2019.dta", clear

  3 . drop residualDM beta_DM residualDM_Total_exp beta_DM_Total_exp residualDM_perc_MS beta
    > _DM_perc_MS residualDM_exp_capita beta_DM_exp_capita residualDM_exp_capita_pac beta_DM
    > _exp_capita_pac residualHTN beta_HTN residualHTN_Total_exp beta_HTN_Total_exp residual
    > HTN_perc_MS beta_HTN_perc_MS residualHTN_exp_capita beta_HTN_exp_capita residualHTN_ex
    > p_capita_pac beta_HTN_exp_capita_pac

  4 . do "C:\Users\Owner\AppData\Local\Temp\STD00000000.tmp"

  5 . ***************************************************************************************
    > ********************
  6 . ****** MODELS FOR DISPENSINGS (EXCLUDED INTERVENTION TRANSITION - 2 MONTHS FOR BOTH IN
    > TERVENTIONS ********
  7 . ***************************************************************************************
    > ********************
  8 .
  9 . *******************************
 10 . **** Diabetes Overall  ********
 11 . *******************************
 12 .
 13 . ***********************************
 14 . **** Number of individuals ********
 15 . ***********************************
 16 .
 17 . ***prais DM_ind_count_d Tempo level_AFP_II_a trend_AFP_II_a level_SNP_a trend_SNP_a, r
    > hotype(dw)
 18 .
 19 . prais DM_ind_count_d Tempo level_AFP_II_a trend_AFP_II_a level_SNP_a trend_SNP_a, corc

Number of gaps in sample:    2
(note: computations for rho restarted at each gap)

Iteration 0:  rho =  0.0000
Iteration 1:  rho =  0.1104
Iteration 2:  rho =  0.1397
Iteration 3:  rho =  0.1454
Iteration 4:  rho =  0.1466
Iteration 5:  rho =  0.1468
Iteration 6:  rho =  0.1469
Iteration 7:  rho =  0.1469
Iteration 8:  rho =  0.1469
Iteration 9:  rho =  0.1469
```

Cochrane-Orcutt AR(1) regression -- iterated estimates

Source	SS	df	MS
Model	**867.573386**	**5**	**173.514677**
Residual	**5.37929793**	**62**	**.08676287**
Total	**872.952684**	**67**	**13.0291445**

Number of obs	=	**68**
F(5, 62)	=	**1999.87**
Prob > F	=	**0.0000**
R-squared	=	**0.9938**
Adj R-squared	=	**0.9933**
Root MSE	=	**.29456**

DM_ind_count_d	Coef.	Std. Err.	t	P>|t|	[95% Conf.	Interval]
Tempo	**.1211879**	**.0076632**	**15.81**	**0.000**	**.1058693**	**.1365064**
level_AFP_II_a	**-1.737563**	**.2262778**	**-7.68**	**0.000**	**-2.189886**	**-1.28524**
trend_AFP_II_a	**-.1092219**	**.0163667**	**-6.67**	**0.000**	**-.1419385**	**-.0765054**
level_SNP_a	**4.580765**	**.2473427**	**18.52**	**0.000**	**4.086334**	**5.075196**
trend_SNP_a	**.3202013**	**.0197081**	**16.25**	**0.000**	**.2808054**	**.3595973**
_cons	**.598661**	**.1395337**	**4.29**	**0.000**	**.3197371**	**.8775849**
rho	**.1468856**					

```
Durbin-Watson statistic (original)      1.356001
Durbin-Watson statistic (transformed)   2.224562

20 .
21 .
22 . **GENERATE PREDICTIONS FROM MODEL
23 . predict residual, residual
   (4 missing values generated)

24 . predict beta
   (option xb assumed; fitted values)
   (4 missing values generated)

25 .
26 . rename residual residualDM

27 . rename beta beta_DM

28 .
29 .
30 . ********************************************************************************
31 .
32 . ******************************************
33 . **** Diabetes Total expenditure   ********
34 . ******************************************
35 .
36 . prais DM_Total_exp Tempo level_AFP_II_a trend_AFP_II_a level_SNP_a trend_SNP_a, corc

   Number of gaps in sample:    2
   (note: computations for rho restarted at each gap)

   Iteration 0:  rho =  0.0000
   Iteration 1:  rho =  0.6516
   Iteration 2:  rho =  0.6578
   Iteration 3:  rho =  0.6588
   Iteration 4:  rho =  0.6590
   Iteration 5:  rho =  0.6590
   Iteration 6:  rho =  0.6590
   Iteration 7:  rho =  0.6590

   Cochrane-Orcutt AR(1) regression -- iterated estimates
```

```
      Source |       SS       df       MS              Number of obs =        68
-------------+------------------------------           F(  5,    62) =     75.17
       Model |  2.2418e+14     5  4.4836e+13           Prob > F      =    0.0000
    Residual |  3.6983e+13    62  5.9650e+11           R-squared     =    0.8584
-------------+------------------------------           Adj R-squared =    0.8470
       Total |  2.6116e+14    67  3.8980e+12           Root MSE      =   7.7e+05

------------------------------------------------------------------------------
DM_Total_exp |      Coef.   Std. Err.      t    P>|t|     [95% Conf. Interval]
-------------+----------------------------------------------------------------
       Tempo |   356952.8   50268.66     7.10   0.000     256467.2    457438.4
level_AFP_II_a |   -4417562    1565916    -2.82   0.006     -7547784    -1287340
trend_AFP_II_a |  -367883.2     107361    -3.43   0.001    -582494.8   -153271.6
  level_SNP_a |    2904400    1630404     1.78   0.080    -354731.6     6163532
  trend_SNP_a |   543104.9   129279.8     4.20   0.000     284678.3    801531.5
       _cons |    2117644   994729.4     2.13   0.037     129208.1     4106080
-------------+----------------------------------------------------------------
         rho |   .6589963
------------------------------------------------------------------------------
Durbin-Watson statistic (original)      0.677711
Durbin-Watson statistic (transformed)   2.121174

37 .
38 . **GENERATE PREDICTIONS FROM MODEL
39 . predict residual, residual
   (4 missing values generated)

40 . predict beta
   (option xb assumed; fitted values)
   (4 missing values generated)

41 .
42 . rename residual residualDM_Total_exp

43 . rename beta beta_DM_Total_exp

44 .
45 .
46 . ***********************************
47 . **** Diabetes Percent MS   ********
48 . ***********************************
49 .
50 . ***prais DM_perc_MS Tempo level_AFP_II_a trend_AFP_II_a level_SNP_a trend_SNP_a, rhoty
   > pe(dw)
51 .
52 .
53 . prais DM_perc_MS Tempo level_AFP_II_a trend_AFP_II_a level_SNP_a trend_SNP_a, corc

   Number of gaps in sample:    2
   (note: computations for rho restarted at each gap)

   Iteration 0:  rho =  0.0000
   Iteration 1:  rho =  0.6562
   Iteration 2:  rho =  0.6607
   Iteration 3:  rho =  0.6614
   Iteration 4:  rho =  0.6615
   Iteration 5:  rho =  0.6615
   Iteration 6:  rho =  0.6615
   Iteration 7:  rho =  0.6615

   Cochrane-Orcutt AR(1) regression -- iterated estimates
```

Source	SS	df	MS
Model	**2104.66702**	**5**	**420.933404**
Residual	**13.9744556**	**62**	**.225394445**
Total	**2118.64147**	**67**	**31.6215145**

```
Number of obs =          68
F(  5,    62) =     1867.54
Prob > F      =      0.0000
R-squared     =      0.9934
Adj R-squared =      0.9929
Root MSE      =      .47476
```

DM_perc_MS	Coef.	Std. Err.	t	P>|t|	[95% Conf.	Interval]
Tempo	**-.3237033**	**.0311265**	**-10.40**	**0.000**	**-.3859244**	**-.2614823**
level_AFP_II_a	**-13.93192**	**.9703073**	**-14.36**	**0.000**	**-15.87154**	**-11.99231**
trend_AFP_II_a	**.3279685**	**.0664783**	**4.93**	**0.000**	**.1950803**	**.4608567**
level_SNP_a	**42.86989**	**1.009733**	**42.46**	**0.000**	**40.85146**	**44.88832**
trend_SNP_a	**-.0042652**	**.0800505**	**-0.05**	**0.958**	**-.1642838**	**.1557534**
_cons	**81.32671**	**.6165452**	**131.91**	**0.000**	**80.09425**	**82.55916**
rho	**.6614733**					

```
Durbin-Watson statistic (original)      0.665508
Durbin-Watson statistic (transformed)   1.778373

 54 .
 55 .
 56 . **GENERATE PREDICTIONS FROM MODEL
 57 . predict residual, residual
    (4 missing values generated)

 58 . predict beta
    (option xb assumed; fitted values)
    (4 missing values generated)

 59 .
 60 . rename residual residualDM_perc_MS

 61 . rename beta beta_DM_perc_MS

 62 .
 63 .
 64 .
 65 . ********************************************
 66 . **** Diabetes Expenditure Percapita  ******
 67 . ********************************************
 68 .
 69 . ***prais DM_exp_capita Tempo level_AFP_II_a trend_AFP_II_a level_SNP_a trend_SNP_a, rh
    > otype(dw)
 70 .
 71 . prais DM_exp_capita Tempo level_AFP_II_a trend_AFP_II_a level_SNP_a trend_SNP_a, corc

    Number of gaps in sample:    2
    (note: computations for rho restarted at each gap)

    Iteration 0:  rho =  0.0000
    Iteration 1:  rho =  0.7781
    Iteration 2:  rho =  0.7826
    Iteration 3:  rho =  0.7833
    Iteration 4:  rho =  0.7834
    Iteration 5:  rho =  0.7834
    Iteration 6:  rho =  0.7834
    Iteration 7:  rho =  0.7834

    Cochrane-Orcutt AR(1) regression -- iterated estimates
```

Source	SS	df	MS
Model	**162.15896**	**5**	**32.4317921**
Residual	**221.301248**	**62**	**3.56937497**
Total	**383.460208**	**67**	**5.72328669**

Number of obs =	**68**
F(5, 62) =	**9.09**
Prob > F =	**0.0000**
R-squared =	**0.4229**
Adj R-squared =	**0.3763**
Root MSE =	**1.8893**

DM_exp_capita	Coef.	Std. Err.	t	P>|t|	[95% Conf.	Interval]
Tempo	**-.1101953**	**.1936078**	**-0.57**	**0.571**	**-.4972117**	**.2768212**
level_AFP_II_a	**5.96539**	**6.395247**	**0.93**	**0.355**	**-6.818527**	**18.74931**
trend_AFP_II_a	**-.2036914**	**.4134967**	**-0.49**	**0.624**	**-1.03026**	**.6228768**
level_SNP_a	**-15.8297**	**6.421462**	**-2.47**	**0.016**	**-28.66602**	**-2.993385**
trend_SNP_a	**.4941281**	**.497916**	**0.99**	**0.325**	**-.5011918**	**1.489448**
_cons	**33.06701**	**4.129037**	**8.01**	**0.000**	**24.81318**	**41.32083**
rho	**.7834166**					

```
Durbin-Watson statistic (original)      0.462208
Durbin-Watson statistic (transformed)   1.912641

72 .
73 . **GENERATE PREDICTIONS FROM MODEL
74 . predict residual, residual
   (4 missing values generated)

75 . predict beta
   (option xb assumed; fitted values)
   (4 missing values generated)

76 .
77 . rename residual residualDM_exp_capita

78 . rename beta beta_DM_exp_capita

79 .
80 .
81 .
82 . *********************************************
83 . **** Diabetes Out of pocket payment  ********
84 . *********************************************
85 .
86 . ***prais DM_exp_capita_pac Tempo level_AFP_II_a trend_AFP_II_a level_SNP_a trend_SNP_a
   > , rhotype(dw)
87 .
88 . prais DM_exp_capita_pac Tempo level_AFP_II_a trend_AFP_II_a level_SNP_a trend_SNP_a, c
   > orc

   Number of gaps in sample:    2
   (note: computations for rho restarted at each gap)

   Iteration 0:  rho =  0.0000
   Iteration 1:  rho =  0.7446
   Iteration 2:  rho =  0.7488
   Iteration 3:  rho =  0.7495
   Iteration 4:  rho =  0.7496
   Iteration 5:  rho =  0.7496
   Iteration 6:  rho =  0.7496
   Iteration 7:  rho =  0.7496

   Cochrane-Orcutt AR(1) regression -- iterated estimates
```

Source	SS	df	MS
Model	**111.477534**	**5**	**22.2955068**
Residual	**13.1647633**	**62**	**.212334892**
Total	**124.642297**	**67**	**1.86033279**

Number of obs =	**68**
F(5, 62) =	**105.00**
Prob > F =	**0.0000**
R-squared =	**0.8944**
Adj R-squared =	**0.8859**
Root MSE =	**.4608**

DM_exp_capit~c	Coef.	Std. Err.	t	P>|t|	[95% Conf.	Interval]
Tempo	**.0732483**	**.0408471**	**1.79**	**0.078**	**-.0084039**	**.1549004**
level_AFP_II_a	**6.635445**	**1.319448**	**5.03**	**0.000**	**3.997906**	**9.272983**
trend_AFP_II_a	**-.2079542**	**.0872389**	**-2.38**	**0.020**	**-.3823423**	**-.0335662**
level_SNP_a	**-12.26695**	**1.341063**	**-9.15**	**0.000**	**-14.94769**	**-9.5862**
trend_SNP_a	**.134706**	**.1050495**	**1.28**	**0.205**	**-.075285**	**.344697**
_cons	**6.251089**	**.8477794**	**7.37**	**0.000**	**4.556403**	**7.945776**
rho	**.7496187**					

```
Durbin-Watson statistic (original)      0.514678
Durbin-Watson statistic (transformed)   1.793323

 89 .
 90 . **GENERATE PREDICTIONS FROM MODEL
 91 . predict residual, residual
    (4 missing values generated)

 92 . predict beta
    (option xb assumed; fitted values)
    (4 missing values generated)

 93 .
 94 . rename residual residualDM_exp_capita_pac

 95 . rename beta beta_DM_exp_capita_pac

 96 .
 97 .
 98 . ******************************
 99 . **** Hypertension Overall  ****
100 . ******************************
101 .
102 . ********************************************
103 . **** Hypertension number of individuals  ****
104 . ********************************************
105 .
106 . ***prais HTN_ind_count_d Tempo level_AFP_II_a trend_AFP_II_a level_SNP_a trend_SNP_a,
    > rhotype(dw)
107 .
108 . prais HTN_ind_count_d Tempo level_AFP_II_a trend_AFP_II_a level_SNP_a trend_SNP_a, cor
    > c

    Number of gaps in sample:    2
    (note: computations for rho restarted at each gap)

    Iteration 0:  rho =  0.0000
    Iteration 1:  rho =  0.1738
    Iteration 2:  rho =  0.2018
    Iteration 3:  rho =  0.2076
    Iteration 4:  rho =  0.2088
    Iteration 5:  rho =  0.2091
    Iteration 6:  rho =  0.2092
    Iteration 7:  rho =  0.2092
    Iteration 8:  rho =  0.2092
    Iteration 9:  rho =  0.2092
```

```
Cochrane-Orcutt AR(1) regression -- iterated estimates

      Source |       SS       df       MS              Number of obs =        68
-------------+------------------------------           F(  5,    62) =   1144.97
       Model |  6398.46563     5  1279.69313           Prob > F      =    0.0000
    Residual |  69.2950165    62  1.11766156           R-squared     =    0.9893
-------------+------------------------------           Adj R-squared =    0.9884
       Total |  6467.76065    67   96.533741           Root MSE      =    1.0572

------------------------------------------------------------------------------
HTN_ind_coun~d |      Coef.   Std. Err.      t    P>|t|     [95% Conf. Interval]
---------------+--------------------------------------------------------------
         Tempo |   .3124578   .0296719    10.53   0.000     .2531445    .3717712
level_AFP_II_a |  -4.876448   .8783862    -5.55   0.000    -6.632317   -3.120579
trend_AFP_II_a |  -.2978411   .0633717    -4.70   0.000    -.4245193    -.171163
   level_SNP_a |   15.49915   .9574851    16.19   0.000     13.58517    17.41314
   trend_SNP_a |   .8528452   .0763096    11.18   0.000     .7003045    1.005386
         _cons |   .7329149   .5427092     1.35   0.182    -.3519452    1.817775
---------------+--------------------------------------------------------------
           rho |   .2092097
------------------------------------------------------------------------------
Durbin-Watson statistic (original)      1.266337
Durbin-Watson statistic (transformed)   2.355599

109 .
110 . **GENERATE PREDICTIONS FROM MODEL
111 . predict residual, residual
    (4 missing values generated)

112 . predict beta
    (option xb assumed; fitted values)
    (4 missing values generated)

113 .
114 . rename residual residualHTN

115 . rename beta beta_HTN

116 .
117 .
118 . ******************************************
119 . **** Hypertension Total expenditure   ****
120 . ******************************************
121 .
122 . ***prais HTN_Total_exp Tempo level_AFP_II_a trend_AFP_II_a level_SNP_a trend_SNP_a, rh
    > otype(dw)
123 .
124 . prais HTN_Total_exp Tempo level_AFP_II_a trend_AFP_II_a level_SNP_a trend_SNP_a, corc

    Number of gaps in sample:    2
    (note: computations for rho restarted at each gap)

    Iteration 0:  rho =  0.0000
    Iteration 1:  rho =  0.7402
    Iteration 2:  rho =  0.7520
    Iteration 3:  rho =  0.7579
    Iteration 4:  rho =  0.7610
    Iteration 5:  rho =  0.7628
    Iteration 6:  rho =  0.7638
    Iteration 7:  rho =  0.7644
    Iteration 8:  rho =  0.7647
    Iteration 9:  rho =  0.7649
    Iteration 10:  rho =  0.7650
    Iteration 11:  rho =  0.7651
    Iteration 12:  rho =  0.7652
    Iteration 13:  rho =  0.7652
    Iteration 14:  rho =  0.7652
```

```
Iteration 15:  rho =  0.7652
Iteration 16:  rho =  0.7652
Iteration 17:  rho =  0.7652
Iteration 18:  rho =  0.7652
Iteration 19:  rho =  0.7652
Iteration 20:  rho =  0.7652

Cochrane-Orcutt AR(1) regression -- iterated estimates

      Source |       SS       df       MS              Number of obs =        68
-------------+------------------------------           F(  5,    62) =     44.35
       Model |  1.6826e+15     5  3.3651e+14           Prob > F      =    0.0000
    Residual |  4.7039e+14    62  7.5869e+12           R-squared     =    0.7815
-------------+------------------------------           Adj R-squared =    0.7639
       Total |  2.1530e+15    67  3.2134e+13           Root MSE      =    2.8e+06

------------------------------------------------------------------------------
HTN_Total_exp |      Coef.   Std. Err.      t    P>|t|     [95% Conf. Interval]
-------------+----------------------------------------------------------------
       Tempo |    1271090   260380.3     4.88   0.000     750597.4     1791583
level_AFP_II_a |  -1.94e+07    8490509    -2.28   0.026    -3.63e+07    -2399550
trend_AFP_II_a |   -1272637   556105.6    -2.29   0.026     -2384276   -160997.6
 level_SNP_a |   3.46e+07    8583498     4.03   0.000     1.75e+07    5.18e+07
 trend_SNP_a |   738447.5   669639.9     1.10   0.274    -600143.5     2077039
       _cons |   199490.1    5467461     0.04   0.971    -1.07e+07    1.11e+07
-------------+----------------------------------------------------------------
         rho |   .7652123
------------------------------------------------------------------------------
Durbin-Watson statistic (original)      0.490229
Durbin-Watson statistic (transformed)   2.196999

125 .
126 . **GENERATE PREDICTIONS FROM MODEL
127 . predict residual, residual
    (4 missing values generated)

128 . predict beta
    (option xb assumed; fitted values)
    (4 missing values generated)

129 .
130 . rename residual residualHTN_Total_exp

131 . rename beta beta_HTN_Total_exp

132 .
133 .
134 . ************************************
135 . **** Hypertension Percent MS   ****
136 . ************************************
137 .
138 . ***prais HTN_perc_MS Tempo level_AFP_II_a trend_AFP_II_a level_SNP_a trend_SNP_a, rhot
    > ype(dw)
139 .
140 . prais HTN_perc_MS Tempo level_AFP_II_a trend_AFP_II_a level_SNP_a trend_SNP_a, corc

Number of gaps in sample:   2
(note: computations for rho restarted at each gap)

Iteration 0:  rho =  0.0000
Iteration 1:  rho =  0.5829
Iteration 2:  rho =  0.5819
Iteration 3:  rho =  0.5817
Iteration 4:  rho =  0.5817
Iteration 5:  rho =  0.5817
Iteration 6:  rho =  0.5817
```

Cochrane-Orcutt AR(1) regression -- iterated estimates

Source	SS	df	MS
Model	**1733.04753**	**5**	**346.609507**
Residual	**34.8793208**	**62**	**.56256969**
Total	**1767.92685**	**67**	**26.386968**

Number of obs =	**68**
F(5, 62) =	**616.12**
Prob > F =	**0.0000**
R-squared =	**0.9803**
Adj R-squared =	**0.9787**
Root MSE =	**.75005**

HTN_perc_MS	Coef.	Std. Err.	t	P>|t|	[95% Conf.	Interval]
Tempo	**-.0508355**	**.0397986**	**-1.28**	**0.206**	**-.1303917**	**.0287208**
level_AFP_II_a	**-16.3997**	**1.218289**	**-13.46**	**0.000**	**-18.83502**	**-13.96437**
trend_AFP_II_a	**.2845781**	**.0849996**	**3.35**	**0.001**	**.1146662**	**.45449**
level_SNP_a	**29.46988**	**1.286212**	**22.91**	**0.000**	**26.89878**	**32.04098**
trend_SNP_a	**-.2337426**	**.1023531**	**-2.28**	**0.026**	**-.4383436**	**-.0291417**
_cons	**83.41422**	**.7680585**	**108.60**	**0.000**	**81.87889**	**84.94954**
rho	**.581715**					

```
Durbin-Watson statistic (original)      0.751780
Durbin-Watson statistic (transformed)   1.819471

141 .
142 .
143 . **GENERATE PREDICTIONS FROM MODEL
144 . predict residual, residual
    (4 missing values generated)

145 . predict beta
    (option xb assumed; fitted values)
    (4 missing values generated)

146 .
147 . rename residual residualHTN_perc_MS

148 . rename beta beta_HTN_perc_MS

149 .
150 .
151 . ********************************************
152 . **** Hypertension Expenditure Percapita  ***
153 . ********************************************
154 .
155 . ***prais HTN_exp_capita Tempo level_AFP_II_a trend_AFP_II_a level_SNP_a trend_SNP_a, r
    > hotype(dw)
156 .
157 . prais HTN_exp_capita Tempo level_AFP_II_a trend_AFP_II_a level_SNP_a trend_SNP_a, corc

    Number of gaps in sample:    2
    (note: computations for rho restarted at each gap)

    Iteration 0:  rho =  0.0000
    Iteration 1:  rho =  0.7843
    Iteration 2:  rho =  0.7873
    Iteration 3:  rho =  0.7876
    Iteration 4:  rho =  0.7876
    Iteration 5:  rho =  0.7876
    Iteration 6:  rho =  0.7876
```

Cochrane-Orcutt AR(1) regression -- iterated estimates

Source	SS	df	MS
Model	**196.21675**	**5**	**39.24335**
Residual	**617.084564**	**62**	**9.95297684**
Total	**813.301314**	**67**	**12.1388256**

Number of obs =	**68**
F(5, 62) =	**3.94**
Prob > F =	**0.0036**
R-squared =	**0.2413**
Adj R-squared =	**0.1801**
Root MSE =	**3.1548**

HTN_exp_capita	Coef.	Std. Err.	t	P>|t|	[95% Conf.	Interval]
Tempo	**.0105722**	**.3296641**	**0.03**	**0.975**	**-.6484168**	**.6695613**
level_AFP_II_a	**4.511183**	**10.92558**	**0.41**	**0.681**	**-17.32874**	**26.35111**
trend_AFP_II_a	**-.399688**	**.7040781**	**-0.57**	**0.572**	**-1.80712**	**1.007744**
level_SNP_a	**-14.59747**	**10.95229**	**-1.33**	**0.187**	**-36.49078**	**7.295838**
trend_SNP_a	**.4379438**	**.8478223**	**0.52**	**0.607**	**-1.256829**	**2.132717**
_cons	**36.42778**	**7.058262**	**5.16**	**0.000**	**22.31851**	**50.53704**
rho	**.7875988**					

Durbin-Watson statistic (original)   **0.445208**
Durbin-Watson statistic (transformed) **1.897956**

```
158 .
159 . **GENERATE PREDICTIONS FROM MODEL
160 . predict residual, residual
    (4 missing values generated)

161 . predict beta
    (option xb assumed; fitted values)
    (4 missing values generated)

162 .
163 . rename residual residualHTN_exp_capita

164 . rename beta beta_HTN_exp_capita

165 .
166 .
167 . *********************************************
168 . **** Hypertension Out of pocket payment *****
169 . *********************************************
170 .
171 . ***prais HTN_exp_capita_pac Tempo level_AFP_II_a trend_AFP_II_a level_SNP_a trend_SNP_
    > a, rhotype(dw)
172 .
173 . prais HTN_exp_capita_pac Tempo level_AFP_II_a trend_AFP_II_a level_SNP_a trend_SNP_a,
    > corc

    Number of gaps in sample:    2
    (note: computations for rho restarted at each gap)

    Iteration 0:  rho =  0.0000
    Iteration 1:  rho =  0.7381
    Iteration 2:  rho =  0.7390
    Iteration 3:  rho =  0.7391
    Iteration 4:  rho =  0.7391
    Iteration 5:  rho =  0.7391
    Iteration 6:  rho =  0.7391

    Cochrane-Orcutt AR(1) regression -- iterated estimates
```

```
      Source |       SS       df       MS              Number of obs =        68
-------------+------------------------------           F(  5,    62) =     40.69
       Model |  87.8378349     5  17.567567            Prob > F      =    0.0000
    Residual |  26.7666002    62  .431719358           R-squared     =    0.7664
-------------+------------------------------           Adj R-squared =    0.7476
       Total |  114.604435    67  1.71051396           Root MSE      =    .65705

------------------------------------------------------------------------------
HTN_exp_capi~c |      Coef.   Std. Err.      t    P>|t|     [95% Conf. Interval]
---------------+----------------------------------------------------------------
         Tempo |   .0083613   .0558979     0.15   0.882    -.1033771    .1200996
level_AFP_II_a |   7.625822   1.795443     4.25   0.000     4.036783    11.21486
trend_AFP_II_a |   -.214154   .1193837    -1.79   0.078    -.4527986    .0244906
   level_SNP_a |  -9.655057   1.831035    -5.27   0.000    -13.31525    -5.99487
   trend_SNP_a |   .2057927    .143757     1.43   0.157    -.0815735    .4931589
         _cons |   6.289115   1.151936     5.46   0.000     3.986427    8.591802
---------------+----------------------------------------------------------------
           rho |   .7391103
--------------------------------------------------------------------------------
Durbin-Watson statistic (original)      0.505836
Durbin-Watson statistic (transformed)   1.870613

174 .
175 .
176 . **GENERATE PREDICTIONS FROM MODEL
177 . predict residual, residual
    (4 missing values generated)

178 . predict beta
    (option xb assumed; fitted values)
    (4 missing values generated)

179 .
180 . rename residual residualHTN_exp_capita_pac

181 . rename beta beta_HTN_exp_capita_pac

182 .
    end of do-file

183 .
```
